# Externally validated explainable machine learning for postoperative recurrence prediction in early-stage NSCLC

**DOI:** 10.3389/fonc.2026.1913341

**Published:** 2026-09-11

**Authors:** Fatih Kemik, Hayri Kağan Gören, Bahadır Köylü, Cevat İlteriş Kıkılı, Nazan Demir, Berna Karataş, Salih Duman, Kadir Burak Özer, Suat Erus, Berker Özkan, Serhan Tanju, Şükrü Dilege, Fatih Selçukbiricik

**Affiliations:** 1 Department of Medical Oncology, Koç University School of Medicine, Istanbul, Türkiye; 2 Department of Internal Medicine, Koç University School of Medicine, Istanbul, Türkiye; 3 Department of Thoracic Surgery, Istanbul University, Istanbul Faculty of Medicine, Istanbul, Türkiye; 4 Department of Thoracic Surgery, Koç University School of Medicine, Istanbul, Türkiye

**Keywords:** early-stage lung cancer, external validation, machine learning, postoperative recurrence, random forest

## Abstract

**Background:**

Postoperative recurrence remains a major challenge in early-stage non–small cell lung cancer (NSCLC), and pathological TNM staging does not fully capture within-stage heterogeneity. We aimed to develop and externally validate an explainable machine learning model for recurrence prediction after curative resection.

**Methods:**

This retrospective study included 723 patients with stage I–II NSCLC, including 52 recurrences, with independent external validation in a separate cohort (n=50). A Random Forest model using routinely available clinical and pathological variables was developed within a nested cross-validation framework and compared with logistic regression. Performance was evaluated using ROC-AUC, calibration, Decision Curve Analysis, and SHAP-based interpretation.

**Results:**

The Random Forest achieved a mean internal ROC-AUC of 0.70 versus 0.64 for logistic regression, although no formal paired statistical comparison was performed. In an independent case-control external validation cohort with an artificially balanced outcome distribution, the Random Forest achieved an ROC-AUC of 0.68 (95% CI 0.53–0.83); the balanced design precluded assessment of calibration or absolute risk at natural prevalence. Sigmoid recalibration of pooled out-of-fold predictions yielded an apparent Brier score reduction to 0.063, although post-calibration performance was not independently evaluated. Decision Curve Analysis demonstrated positive net clinical benefit across clinically relevant thresholds. SHAP analysis highlighted tumor size, pathological T stage, and STAS among the principal tumor-related predictors, whereas fold-level analysis showed greater stability for tumor size, tumor necrosis, and STAS. Complementary time-to-event analyses accounting for right censoring included 715 patients and 44 recurrence events.

**Conclusion:**

An explainable machine learning model based on routinely available variables demonstrated promising but preliminary discrimination in an independent case-control external validation cohort. Larger consecutive cohorts with natural outcome prevalence are required before clinical application.

## Introduction

Surgical resection remains the cornerstone of curative treatment for patients with early-stage (stage I–II) non–small cell lung cancer (NSCLC) (1). Nevertheless, despite complete resection, a substantial proportion of patients experience postoperative recurrence (2). Although the TNM staging system is indispensable for prognostic assessment and therapeutic decision-making, it does not fully account for the considerable clinical heterogeneity observed among patients within the same pathological stage (3) In early-stage disease in particular, some patients remain recurrence-free for prolonged periods, whereas others develop early locoregional or distant relapse (4). These observations suggest that recurrence dynamics may be influenced not only by tumor burden and anatomical extent, but also by tumor biology and host-related factors (5).

In recent years, multivariable statistical models and machine learning (ML) approaches have increasingly been employed to enable individualized risk prediction. Studies incorporating radiomics, imaging-derived features, or high-dimensional datasets have reported promising discriminatory performance (6, 7). However, many of these models are limited by small sample sizes, lack of independent external validation, or an increased risk of overfitting (8, 9). Moreover, the reliance on complex or non-routinely available data may restrict their integration into real-world clinical practice. Therefore, the development of generalizable and explainable models based on routinely collected clinical and pathological variables represents a clinically meaningful unmet need.

In this study, we aimed to develop and externally validate an explainable machine learning model to predict postoperative recurrence risk in patients with early-stage NSCLC undergoing curative surgical resection, using routinely available clinical and pathological parameters. We hypothesized that recurrence risk could be more accurately estimated through a multidimensional framework that integrates host-reserve–related variables alongside tumor-specific pathological features. The proposed model is intended to serve as a complementary decision-support tool for individualized risk stratification in early-stage NSCLC.

## Materials and methods

### Study design and patient population

This retrospective observational study aims to develop and externally validate a machine learning model to predict postoperative recurrence risk in patients diagnosed with early-stage (Stage I–II) non-small cell lung cancer (NSCLC) who underwent curative surgical resection. The development cohort comprised patients treated at Koç University Hospital. Patients who received neoadjuvant therapy were excluded from the analysis. A total of 723 patients were included in the final analysis. The primary endpoint was defined as disease recurrence, confirmed either radiologically or pathologically during the postoperative follow-up period.

Postoperative surveillance at both centers followed standard NCCN and ESMO guideline recommendations for resected early-stage NSCLC, comprising clinical evaluation and chest CT every 6 months for the first 2 years after surgery, followed by annual chest CT thereafter (1, 10).

To assess the model’s generalizability, an independent external validation cohort of 50 patients from a different center was utilized. To ensure a stable evaluation of discriminative performance, this cohort was designed as a balanced sample, consisting of 25 recurrences and 25 non-recurrences.

For the primary binary-outcome analysis, patients without documented recurrence at their last follow-up were considered recurrence-free for the purposes of binary classification. To additionally account for heterogeneous follow-up and right censoring, patient-level follow-up data were used to construct a time-to-event dataset (n=715; 44 recurrence events). Eight patients with documented recurrence in the binary dataset were excluded from the time-to-event analysis because a sufficiently reliable recurrence date could not be established to define an event time. Patients without recurrence were censored at their last documented follow-up. The binary classification framework was selected *a priori* and retained as the primary analytical approach because the prespecified objective was to develop an explainable, threshold-based clinical decision-support model for recurrence classification. This framework supports probability-based classification, Decision Curve Analysis, and SHAP-based interpretation around a predefined clinical decision threshold. Complementary time-to-event analyses were performed to evaluate the impact of heterogeneous follow-up and right censoring on the primary analysis; their methodology and results are described below under “Time-to-Event Analyses Accounting for Censoring.”

### Time-to-event analyses accounting for censoring

To account for right censoring and heterogeneous follow-up, additional time-to-event analyses were performed using Cox proportional hazards and Random Survival Forest (RSF) models. Both modeling approaches were evaluated using either a TNM-only variable set (pathological T and N stage) or a clinicopathological variable set corresponding to the parsimonious and expanded models. Model performance was assessed at a 36-month prediction horizon using 5-fold cross-validation with out-of-fold predictions and compared using time-dependent area under the receiver operating characteristic curve (time-dependent AUC), Harrell’s concordance index (C-index), and paired bootstrap resampling (1,000 iterations). The primary objective of this study was to develop a clinically applicable binary decision-support model for postoperative recurrence using routinely available clinical and pathological variables. Accordingly, we elected to retain the binary Random Forest classification framework as the primary analysis rather than a survival-based machine learning approach (e.g., Random Survival Forest), because it directly supports threshold-based clinical decision-making, probability calibration, Decision Curve Analysis, and SHAP-based interpretability. The complementary time-to-event analyses described above were performed as corroborating analyses to explicitly evaluate the impact of heterogeneous follow-up and right censoring on the primary modeling framework.

### Data preprocessing and missing data management

All data preprocessing steps were executed in a Python environment using the Scikit-learn library. Pathological T stage and N stage were encoded as ordinal numeric variables to preserve their clinically ordered structure (T1<T2<T3<T4 and N0<N1<N2), whereas binary categorical variables were represented as binary indicators. The ordinal encoding was used as a modeling representation and was not intended to imply that pathological stage variables constitute continuous clinical measurements. Continuous variables were standardized via Z-score normalization.

Missing data patterns were assessed and judged unlikely to be missing completely at random (MCAR). However, the available data did not permit formal distinction between missing at random (MAR) and missing not at random (MNAR), as this generally cannot be established from observed data alone. Missing values were therefore imputed using Multivariate Imputation by Chained Equations (MICE) via the IterativeImputer algorithm under a MAR assumption, consistent with standard practice for prediction modeling. To prevent data leakage, all preprocessing steps including imputation were encapsulated strictly within the cross-validation pipelines. A variance-based feature filtering method was applied to reduce model noise and eliminate non-informative variables. Given the partial biological dependence between tumor size and pathological T stage, a formal collinearity assessment was performed between these two predictors (Pearson correlation and Variance Inflation Factor); both variables were retained in the model, as detailed in the Results.

### Model development

The modeling strategy was designed to compare a linear baseline against a non-linear machine learning approach. Logistic Regression was employed as the reference model and used balanced class weights to address class imbalance. The use of balanced class weights for logistic regression and random undersampling for Random Forest reflects algorithm-specific approaches to class imbalance rather than an assumption that the two strategies are methodologically equivalent. Hyperparameter optimization for the reference model focused on the regularization type (L1/L2) and regularization strength.

The primary predictive model was developed using a Random Forest classifier. To mitigate class imbalance, a RandomUnderSampler strategy was integrated into the training pipeline. To evaluate the impact of this choice on model performance, we additionally trained the Random Forest at the natural cohort prevalence, without undersampling, as a sensitivity analysis. Hyperparameter tuning was performed through Bayesian optimization using the Optuna framework. To constrain model complexity and prevent overfitting, tree depth and the number of features per split were deliberately restricted.

### Validation strategy

A nested cross-validation (Nested CV) approach was adopted to ensure an unbiased estimation of model performance. The inner loop (3-fold stratified CV) was dedicated to hyperparameter optimization, while the outer loop (5-fold stratified CV) was used to evaluate the model’s ability to generalize to unseen data. This architecture ensured that all modeling decisions were made solely based on training data, thereby preventing any information leakage. The complete modeling and validation workflow is illustrated in **Supplementary Figure 1**.

To assess the adequacy of the development sample for prediction model development, a formal minimum sample size calculation was performed according to the framework proposed by Riley et al. (2020), as implemented in the pmsampsize package (11). The calculation was based on the observed outcome prevalence (7.2%) and an anticipated C-statistic of 0.70, considering both a model comprising 10 predictor parameters and the full candidate predictor set of 22 variables evaluated before variance-based filtering.

### Model performance, calibration, and clinical utility

Model discrimination was primarily assessed using the Area Under the Receiver Operating Characteristic curve (ROC-AUC). Sensitivity, specificity, accuracy, and the Youden Index were reported as supplementary performance metrics. The optimal classification threshold was determined on the training data and subsequently applied to the external validation cohort.

Model calibration was evaluated using Brier scores and calibration curves based on out-of-fold (OOF) predictions from the full development cohort at the natural outcome prevalence (52/723 recurrences; 7.2%). *Post-hoc* sigmoid calibration was applied to the pooled OOF predictions after completion of the outer cross-validation procedure. Because the calibration mapping was fitted and subsequently evaluated using the same pooled OOF predictions, the post-calibration Brier score does not constitute a fully independent estimate of calibrated performance and should be interpreted cautiously. Calibration was not assessed in the external validation cohort because its intentionally balanced outcome distribution (50% recurrence) precluded prevalence-valid assessment of probability calibration or absolute risk.

For performance metrics in the external validation set, 95% confidence intervals (CI) were calculated using a 1,000-iteration bootstrap method. The clinical utility of the model was evaluated via Decision Curve Analysis (DCA), comparing the model’s net benefit against “treat-all” and “treat-none” strategies.

### Interpretability and reporting standards

To enhance clinical interpretability, SHapley Additive exPlanations (SHAP) were utilized to evaluate feature contributions at both global and individual levels. The study was designed and reported in accordance with the TRIPOD guidelines (**Supplementary Table 1**), and risk of bias was assessed using the PROBAST framework (**Supplementary Table 2**); the overall PROBAST judgement was high risk of bias, primarily reflecting the Outcome and Analysis domains (9, 12).

### Ethics approval

Ethics approval was obtained from the Koç University Institutional Review Board (IRB No: 2025.431.IRB2.196).

## Results

### Patient characteristics and follow-up

A total of 723 eligible patients were included in the development cohort, of whom 52 had documented postoperative recurrence. The primary endpoint of the study was postoperative disease recurrence, defined as radiologically and/or pathologically confirmed relapse during follow-up. Baseline demographic and clinicopathological characteristics of the development cohort, stratified by recurrence status, are summarized in **Table 1**. Missingness in pulmonary-function variables was pronounced within the recurrence subgroup specifically: FEV1/FVC values were available in 22/52 (42.3%) of patients with recurrence (30/52, 57.7% missing), and DLCO% values were available in 18/52 (34.6%) (34/52, 65.4% missing), substantially exceeding the overall cohort missingness rates for these variables.

**Table 1:** Baseline characteristics of the development cohort, stratified by recurrence status.

Variable	Recurrence (n=52)	No Recurrence (n=671)	Overall (n=723)	p-value
Age (years), mean±SD	66.6 ± 11.7	65.0 ± 9.8	65.2 ± 9.9	0.359
Tumor size (mm), mean±SD	30.7 ± 15.8	22.4 ± 14.5	22.9 ± 14.8	0.0006
DLCO% (n)	76.3 ± 17.3 (n=18)	78.4 ± 19.4 (n=427)	78.3 ± 19.3 (n=445)	0.632
FEV1/FVC% (n)	74.0 ± 9.2 (n=22)	73.3 ± 10.0 (n=453)	73.3 ± 10.0 (n=475)	0.733
Pathological T stage, n (%)				<0.001
T1	10/52 (19.2%)	328/671 (48.9%)	338/723 (46.7%)	
T2	28/52 (53.8%)	254/671 (37.9%)	282/723 (39.0%)	
T3	3/52 (5.8%)	33/671 (4.9%)	36/723 (5.0%)	
T4	11/52 (21.2%)	56/671 (8.3%)	67/723 (9.3%)	
N stage, n (%)				0.50
N0	44/52 (84.6%)	588/671 (87.6%)	632/723 (87.4%)	
N1	6/52 (11.5%)	72/671 (10.7%)	78/723 (10.8%)	
N2	2/52 (3.8%)	11/671 (1.6%)	13/723 (1.8%)	
Lymphatic invasion, n (%)	25/52 (48.1%)	223/671 (33.2%)	248/723 (34.3%)	0.034
Pleural invasion, n (%)	33/52 (63.5%)	250/671 (37.3%)	283/723 (39.1%)	<0.001
Tumor necrosis, n (%)	8/15 (53.3%)	101/390 (25.9%)	109/405 (26.9%)	0.033
STAS positivity, n (%)	14/32 (43.8%)	170/600 (28.3%)	184/632 (29.1%)	0.072

p-values were derived using Welch's t-test for continuous variables, Fisher's exact test for binary categorical variables, and the Fisher–Freeman–Halton exact test (Monte Carlo estimate) for multi-category variables (pathological T stage, N stage). DLCO% and FEV1/FVC% denominators reflect non-missing observations; missingness for these and other variables is reported in the Methods (Data Preprocessing and Missing Data Management). Additional baseline variables (sex, histological cell type, perioperative complications, length of hospital stay) were not available in a format permitting stratified summary at the time of this revision and are noted as a limitation in the Discussion.

A valid event or censoring time required for time-to-event modeling was available for 715 of 723 patients (98.9%). Among the 52 patients with documented recurrence in the binary cohort, 44 had a sufficiently reliable recurrence date to define an event time; the remaining eight were therefore excluded from the time-to-event analyses. Patients without recurrence were censored at their last documented follow-up.

To evaluate the generalizability of the model, an independent external validation cohort consisting of 50 patients was analyzed. In order to provide a stable assessment of discriminative performance, this cohort was intentionally balanced, including 25 patients with recurrence and 25 without recurrence.

### Minimum sample size assessment

According to the framework proposed by Riley et al., a model comprising 10 predictor parameters required an estimated minimum sample size of approximately 2,522 patients (181 recurrence events), whereas the full candidate predictor set of 22 variables required approximately 5,547 patients (398 recurrence events) (11). Accordingly, the available development cohort (723 patients with 52 recurrence events) provided only approximately 13–29% of the recommended minimum number of events, depending on the candidate predictor set considered.

### Model performance - internal validation

In the out-of-fold (OOF) analysis, the optimal classification threshold determined by the Youden index was 0.41. The Youden-optimal threshold (0.41) and the corresponding sensitivity and specificity estimates were derived from the uncalibrated Random Forest probability outputs generated during the OOF analysis of the development cohort. The same threshold was subsequently applied unchanged in the external validation cohort. Sigmoid calibration (see Calibration Analysis) was performed independently to improve probability estimates and did not modify the classification threshold or the reported classification performance metrics.

**Figure 1:**
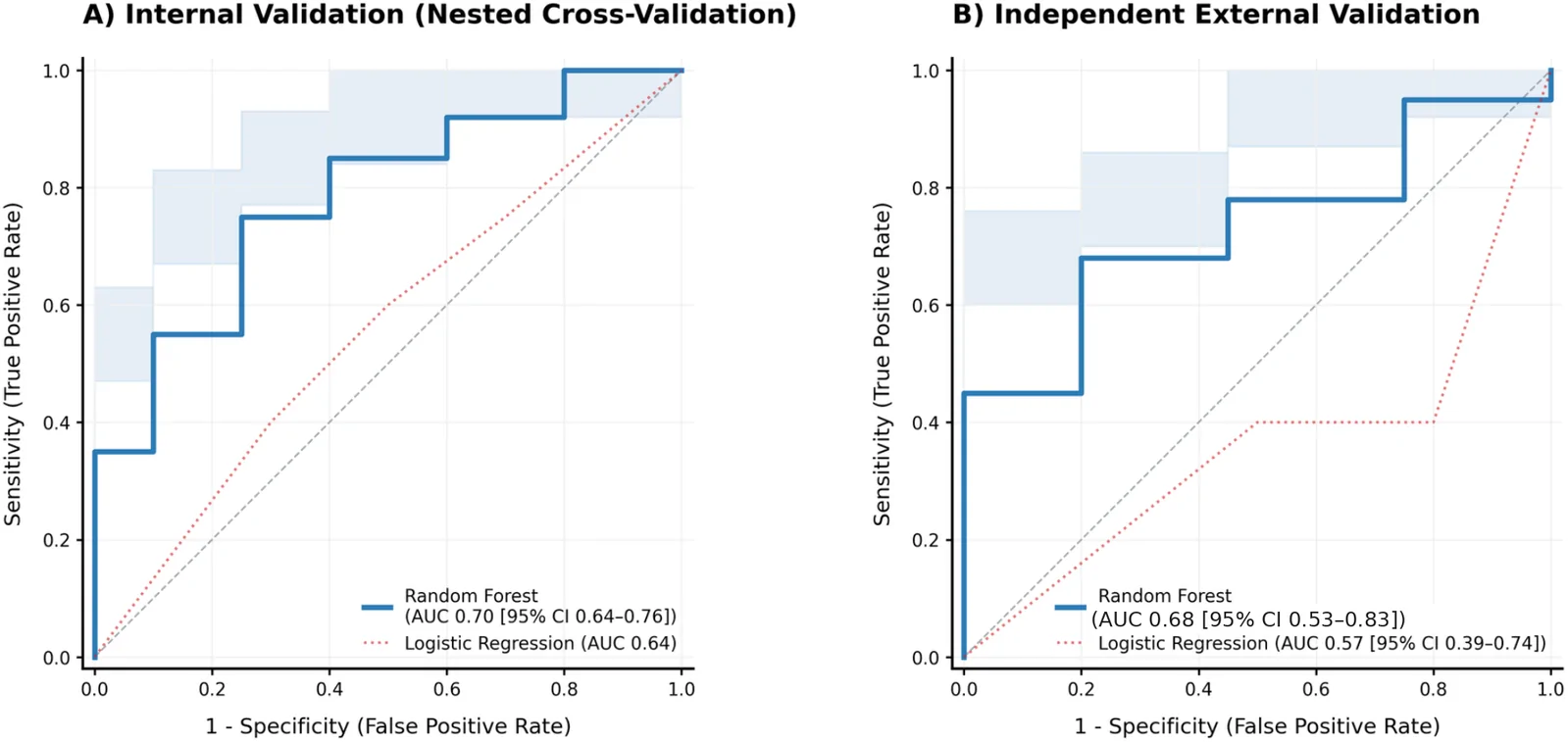
ROC curves for the Random Forest (RF) and Logistic Regression (LR) models in **(A)** internal validation via nested cross-validation (RF AUC: 0.70; LR AUC: 0.64) and **(B)** independent external validation (RF AUC: 0.68, 95% CI: 0.53–0.83; LR AUC: 0.57). Shaded areas represent 95% bootstrap confidence intervals.

The Random Forest (RF) model achieved a mean ROC-AUC of 0.704 in internal validation (**Figure 1A**). At the selected threshold, sensitivity was 73.1%, specificity 63.5%, accuracy 64.2%, and the Youden index 0.366.

The reference Logistic Regression (LR) model demonstrated lower discriminative performance (ROC-AUC: 0.64). Compared with LR, the RF model provided a more balanced performance profile, with higher sensitivity (73.1% vs 65.4%) and specificity (63.5% vs 60.5%).

### External validation performance

In the independent external validation cohort, the RF model demonstrated a similar point estimate of discrimination, with a ROC-AUC of 0.68 (95% CI: 0.53–0.83). However, the wide confidence interval reflects substantial statistical uncertainty, and these findings should therefore be interpreted as preliminary (**Figure 1B**). At the predefined threshold, sensitivity was 76.0% and specificity 60.0%, yielding a Youden index of 0.36.

Bootstrap resampling with 1,000 iterations confirmed the robustness of these estimates. In contrast, the LR model exhibited a substantial decline in discrimination (ROC-AUC: 0.57), primarily driven by a marked reduction in specificity (36.0%), thereby limiting its clinical utility.

To evaluate the impact of undersampling on model performance, we additionally trained the Random Forest at the natural cohort prevalence (52/723), without undersampling, using the same nested cross-validation framework and *post-hoc* sigmoid calibration. This natural-prevalence model achieved an out-of-fold ROC-AUC of 0.71 and an external validation ROC-AUC of 0.65, with a sensitivity of 76.0%, specificity of 36.0%, and Youden index of 0.12, compared with an out-of-fold ROC-AUC of 0.70 and an external validation ROC-AUC of 0.68 for the undersampled primary model. Discrimination was therefore relatively similar between the two approaches, whereas the natural-prevalence model showed lower specificity and a less favorable sensitivity–specificity balance in external validation. *Post-hoc* sigmoid calibration did not improve the Brier score for the natural-prevalence model (0.060 before versus 0.065 after calibration). We do not interpret this comparison as demonstrating that undersampling is intrinsically superior; rather, it provides an empirical assessment of the selected imbalance-handling strategy and suggests that overall discrimination was relatively similar with and without undersampling, while operating characteristics differed.

### Calibration analysis

**Figure 2:**
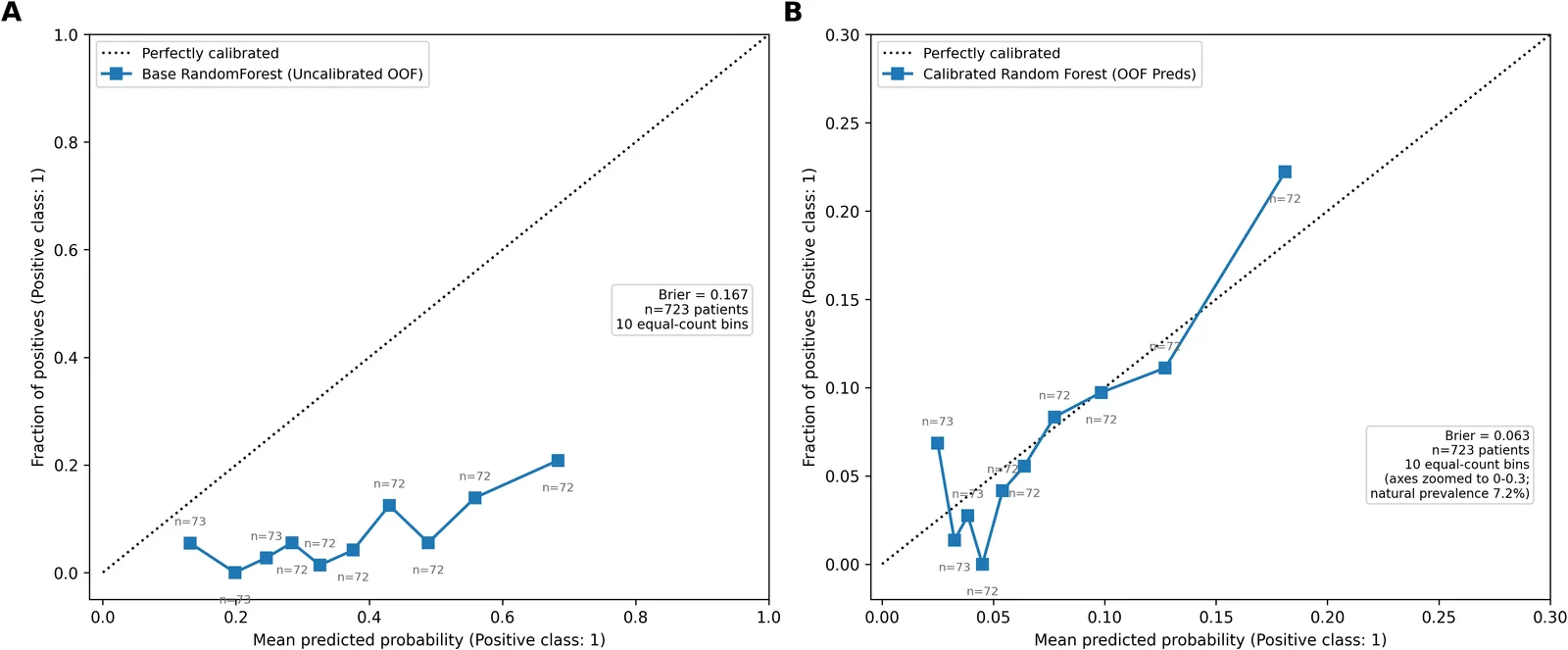
Calibration of the Random Forest model using pooled out-of-fold predictions from the development cohort. **(A)** Calibration of the uncalibrated Random Forest predictions (Brier score = 0.167). **(B)** Calibration after sigmoid recalibration (Brier score = 0.063); the axes are restricted to 0–0.30 to display the calibrated probability range more clearly. Calibration curves were constructed using 10 equal-count bins, with the number of patients in each bin shown adjacent to the corresponding point. The development cohort comprised 723 patients, including 52 recurrence events (7.2%). The sigmoid mapping was fitted to the pooled out-of-fold predictions; therefore, the post-calibration Brier score represents an apparent, same-data estimate and was not independently validated. The dotted diagonal line represents perfect calibration.

Following *post-hoc* sigmoid calibration, the apparent internal Brier score decreased from 0.167 to 0.063 (Brier Skill Score ≈5.5% relative to a null model predicting the base outcome rate). **Figure 2A** shows the uncalibrated Random Forest predictions (Brier score = 0.167), whereas **Figure 2B** shows the corresponding predictions following sigmoid recalibration (Brier score = 0.063). The recalibrated predictions were concentrated within a narrower probability range, with residual deviations between predicted and observed recurrence proportions across individual bins. Because the calibrator was fitted and evaluated on the same pooled OOF predictions, this apparent improvement should be interpreted cautiously and does not represent a fully independent estimate of calibrated performance.

Calibration analysis was not reported for the external validation cohort because the intentionally balanced outcome prevalence (50%) precludes meaningful assessment of absolute risk calibration. The limited sample size (n = 50) represents an additional constraint.

As a further check on the robustness of this finding, in the complementary sensitivity analysis using the Random Forest trained at natural prevalence without undersampling (see External Validation Performance), *post-hoc* sigmoid calibration did not improve the Brier score (0.060 before versus 0.065 after calibration). This result reinforces the cautious interpretation above and indicates that the calibration improvement observed under the primary modeling framework should not be assumed to generalize across modeling choices or deployment prevalence.

### Clinical utility

**Figure 3:**
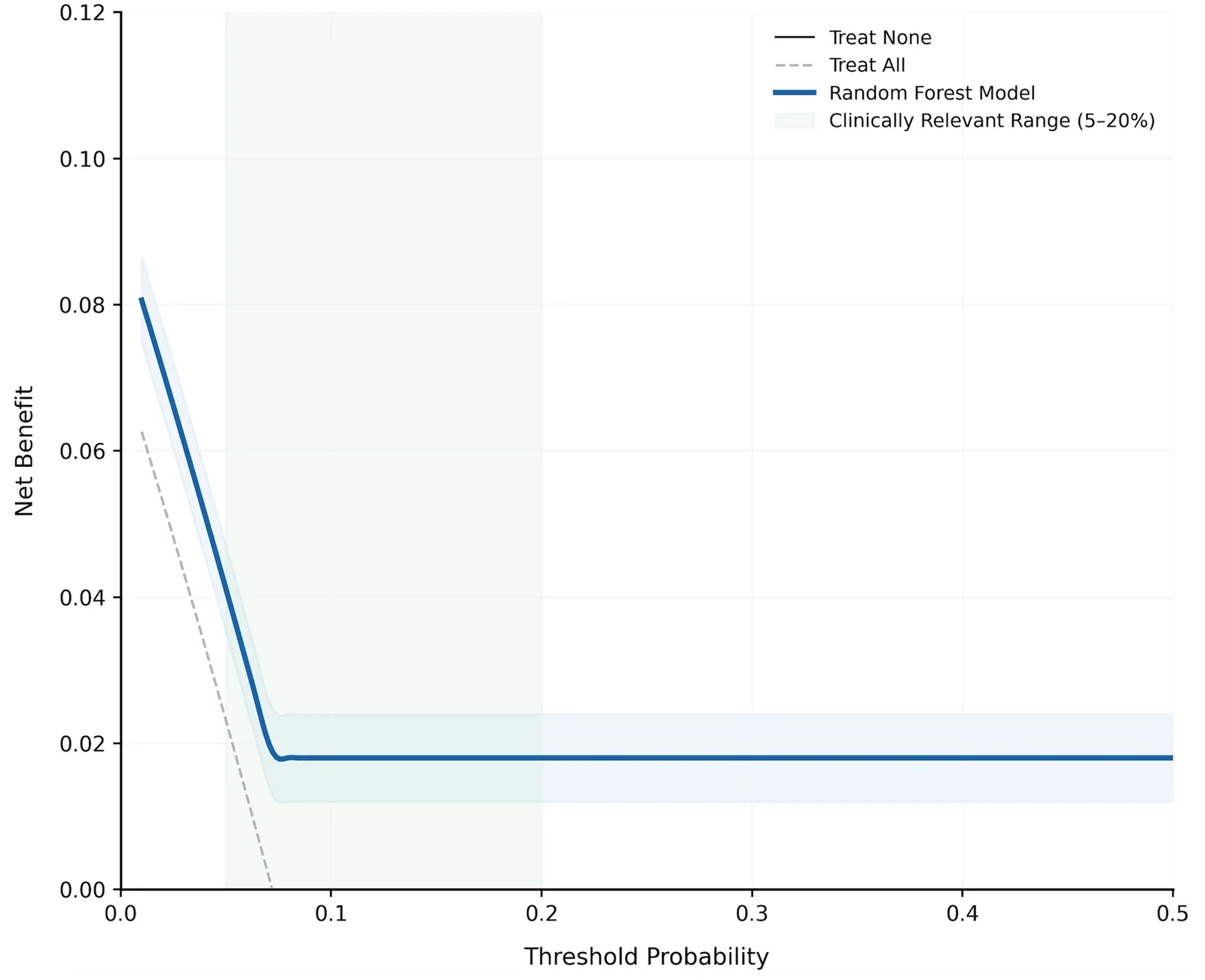
Decision curve analysis of the Random Forest model in internal validation. The model demonstrated positive net clinical benefit over “treat-all” and “treat-none” strategies across threshold probabilities of 5–20% (clinically relevant range, shaded in green).

Decision Curve Analysis demonstrated a modest positive net clinical benefit over both the ‘treat-all’ and ‘treat-none’ strategies, primarily across clinically relevant threshold probabilities of 5–20%, with the net benefit plateauing at approximately 0.018 (**Figure 3**).

### Model interpretability (SHAP)

**Figure 4:**
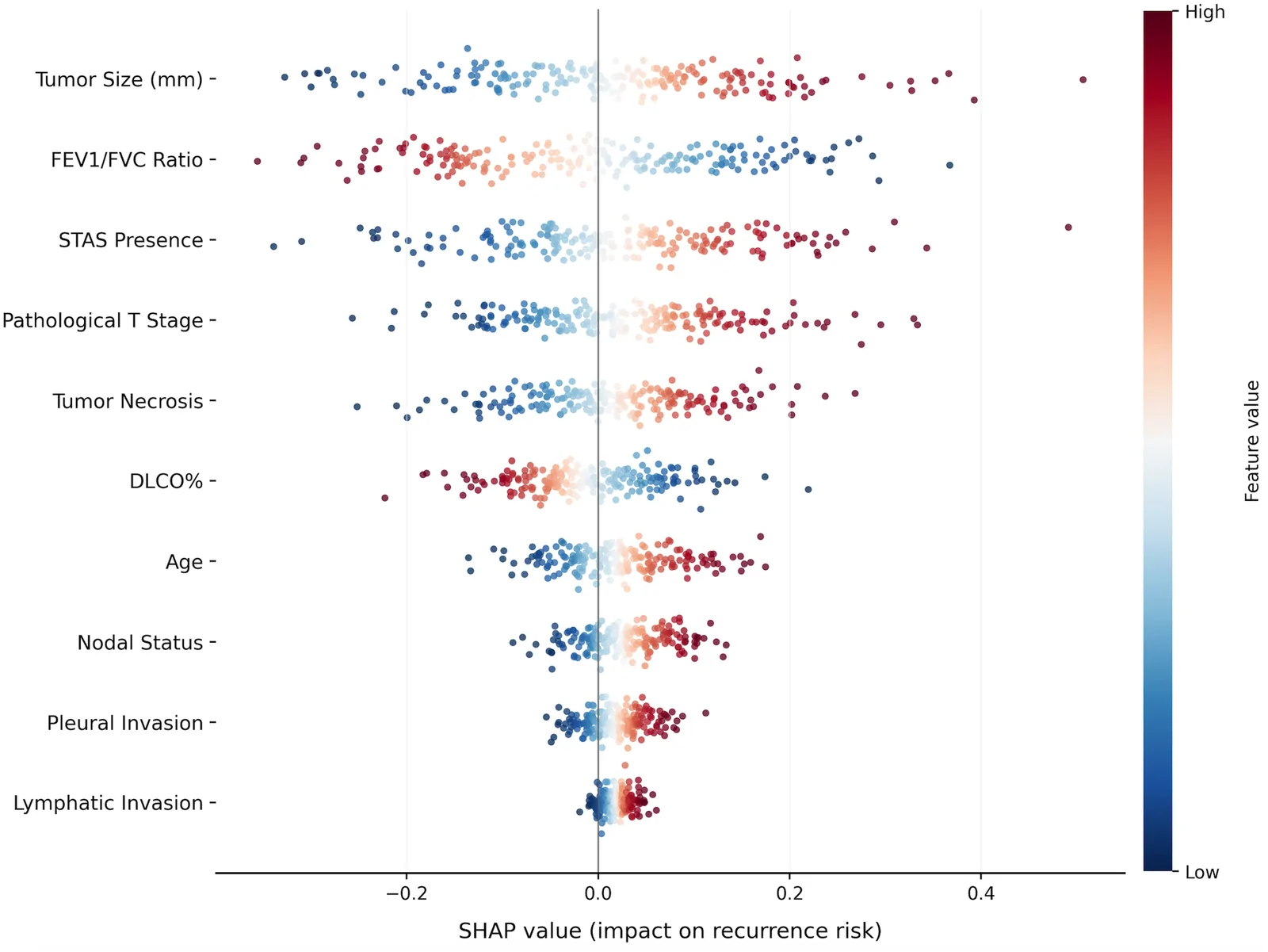
SHAP summary plot showing the contribution of each feature to recurrence risk prediction. Each dot represents one patient; dot color indicates feature value (red = high, blue = low). For pathological T stage, the color gradient reflects the ordinal numerical encoding used in the modeling pipeline (T1<T2<T3<T4) and should not be interpreted as representing a continuous clinical measurement. Features are ranked by mean absolute SHAP value (top = most influential).

Given the partial biological dependence between tumor size and pathological T stage, we assessed collinearity between these two predictors: the Pearson correlation was r = 0.68 (R² = 0.46), corresponding to a Variance Inflation Factor of 1.85 — below conventional thresholds indicative of problematic multicollinearity (VIF >5–10). Both variables were therefore retained. SHAP analysis highlighted tumor size, pathological T stage, and STAS among the principal tumor-related contributors to recurrence prediction (**Figure 4**). Additional variables, including tumor necrosis, DLCO%, age, and nodal status, also contributed to risk estimation. The FEV1/FVC ratio also ranked prominently in the pooled SHAP summary but was not reproducible at the fold level (see Stability of Predictor Importance Across Folds) and, given its substantial missingness among recurrence cases (57.7%), should be regarded as an unreliable, hypothesis-generating signal rather than a robust predictor.

The global SHAP importance ranking highlighted clinically meaningful predictors, and individual-level SHAP explanations enabled visualization of patient-specific risk contributions.

### Comparison with a TNM-only reference model

To evaluate the incremental value of the primary model beyond pathological staging, a TNM-only logistic regression model using pathological T and N stage was developed within the same nested cross-validation and external validation framework. This model achieved a mean internal ROC-AUC of 0.640 (SD 0.123) and an external ROC-AUC of 0.531 (95% CI 0.377–0.686), whereas the primary Random Forest model achieved an external ROC-AUC of 0.68 (95% CI 0.53–0.83). The primary Random Forest model therefore demonstrated numerically higher external discrimination than the TNM-only reference model (0.68 versus 0.53); however, because the paired bootstrap comparison was not independently reverified following confirmation of the final external validation output, we do not report a formal inferential comparison (e.g., paired difference with confidence interval) and instead present this as a descriptive comparison. This numerical difference should not be interpreted as a statistically confirmed incremental improvement over TNM-based staging alone.

### Stability of predictor importance across folds

To assess the robustness of the SHAP-derived feature rankings, permutation importance was additionally calculated separately within each of the five outer cross-validation folds. Tumor size, tumor necrosis, and STAS presence demonstrated consistently high importance across folds. However, tumor necrosis and STAS were available in only 15/52 (28.8%) and 32/52 (61.5%) patients with recurrence, respectively, corresponding to missingness rates of 71.2% and 38.5%; therefore, their apparent fold-level stability should be interpreted cautiously in view of incomplete data availability, particularly for tumor necrosis. In contrast, the FEV1/FVC ratio, despite ranking second in the overall SHAP summary (**Figure 4**), showed near-zero and inconsistent fold-level importance (mean permutation importance −0.001, SD 0.012), ranking eighth among the ten evaluated predictors based on fold-averaged importance. These findings suggest that the apparent importance of FEV1/FVC was not stable across resampling and should therefore be interpreted as hypothesis-generating.

### Time-to-event analyses accounting for censoring

In complementary time-to-event analyses (n=715; 44 recurrence events), discrimination across the six Cox and RSF model configurations was modest and attenuated relative to the primary binary analysis, with Harrell’s C-indices ranging from 0.58 to 0.66 and 36-month time-dependent AUCs from 0.54 to 0.63. These estimates should be interpreted cautiously given the limited number of recurrence events. The TNM-only and parsimonious Cox models and the expanded RSF model showed similar point estimates of discrimination, whereas the expanded Cox model had the lowest point estimates. Complete model-specific estimates with 95% bootstrap confidence intervals are provided in **Supplementary Table 4**.

## Discussion

This study developed and externally validated an explainable machine learning model to predict postoperative recurrence risk in patients with early-stage (Stage I–II) non-small cell lung cancer undergoing curative surgical resection, using only routinely available clinical and pathological variables. The TNM-based staging system does not fully account for the clinical heterogeneity observed within the same stage, underscoring the need for more refined risk stratification in the early setting (13). In this context, logistic regression was used as a linear reference model and compared with the nonlinear Random Forest approach. Sampling strategies and nested cross-validation were implemented to address class imbalance and minimize overfitting. The Random Forest model demonstrated similar point estimates of discrimination in internal validation (ROC-AUC ≈0.70) and external validation (ROC-AUC ≈0.68), although the limited and artificially balanced external cohort warrants cautious interpretation of generalizability.

Although surgical resection is considered curative in early-stage NSCLC, postoperative recurrence remains one of the most critical challenges in clinical practice (14). Systematic reviews have demonstrated that recurrence-free survival (RFS) after surgery varies substantially according to stage. For instance, the 5-year RFS rate in stage I disease is reported to be approximately 80%, whereas it declines progressively with increasing stage (2). In large clinical series, the 5-year RFS for stage II disease has been reported to range between 50% and 60% (15, 16). These “average” estimates also imply considerable heterogeneity within the same stage: while some patients remain recurrence-free for prolonged periods, others experience early locoregional or distant metastatic relapse (17). The literature generally attributes this heterogeneity to two major axes: (i) tumor-related biological and pathological characteristics, including invasion patterns, histologic grade, patterns of spread, and features of the tumor microenvironment; and (ii) host-related factors such as physiological reserve and comorbidity burden, including pulmonary function, systemic inflammatory status, and nutritional or immunonutritional condition (5, 18, 19). Therefore, risk assessment based solely on tumor-centered parameters may not fully capture the heterogeneity observed within the same stage. More comprehensive risk models integrating both tumor and host factors may warrant further investigation for postoperative recurrence risk stratification. Although the primary Random Forest model demonstrated numerically higher discrimination than the TNM-only reference model, this apparent incremental value should be regarded as suggestive rather than firmly established because the external comparison was descriptive and was not supported by a formal inferential comparison.

The prominence of variables such as tumor size, the presence of STAS, and pathologic T stage in the SHAP analysis aligns with the literature, in which recurrence risk in early-stage disease is often explained through a predominantly pathology-driven profile (20). In multicenter early-recurrence modeling studies, pathologic T stage and maximum tumor diameter have consistently been reported among the most influential predictors, supporting the notion that stage- and tumor-burden–related indicators are key determinants of recurrence dynamics (7, 17). With respect to STAS, large-scale meta-analyses have reported a prevalence of approximately 30% and a significant association with worse recurrence-free survival, with pooled hazard ratios generally ranging between 1.8 and 2.0 (21). Nevertheless, given the methodological challenges in STAS assessment — including documented interobserver variability and ongoing debate regarding potential artifacts — variability in pathological interpretation across institutions should be acknowledged (22). In addition, recent work in digital pathology suggests that aggressive features such as pleural invasion, lymphovascular invasion, and tumor necrosis contribute to recurrence risk and that combining these signals with conventional pathological parameters may enhance prognostic discrimination (23, 24). Taken together, our findings support the biological plausibility of the signal captured by routinely available pathological variables, while also suggesting that future multi-layer data integration may provide incremental value. We note that tumor size and pathological T stage were correlated (r = 0.68), although formal assessment did not indicate severe multicollinearity (VIF = 1.85). Nevertheless, this predictor dependence may result in some redistribution of feature attribution between these variables in tree-based SHAP importance estimates; their individual SHAP rankings should therefore not be interpreted as fully independent measures of their respective biological contributions.

Although the machine-learning literature on recurrence prediction in early-stage NSCLC has expanded rapidly, substantial heterogeneity persists with respect to methodological quality and generalizability. Multimodal deep-learning and radiomics-based approaches have reported high discriminatory performance (AUC ~0.86–0.91); however, many studies are characterized by a limited number of participating centers, small external validation cohorts, and an increased risk of overfitting (6, 9). Similarly, models built on extensive laboratory feature sets may demonstrate strong performance, yet the absence of independent and representative external validation remains a frequent limitation (8). Within this context, our study offers a more pragmatic approach to risk stratification by leveraging routinely available clinical and pathological variables, incorporating independent external validation, and maintaining a focus on real-world applicability.

Contemporary methodological standards emphasize that model performance should not be evaluated solely on discriminatory ability (e.g., AUC), but also on calibration, which reflects the reliability of predicted probabilities, and on measures of clinical utility that demonstrate potential impact on decision-making (25). In this regard, the inclusion of calibration assessment and Decision Curve Analysis in our study addresses an important layer that is often insufficiently reported in prognostic modeling research (8). Nevertheless, it should be acknowledged that calibration curves may become unstable in small external validation cohorts, and that differences in event prevalence between derivation and validation samples warrant careful interpretation when translating net clinical benefit into real-world settings (26).

Several limitations of this study should be acknowledged. First, the retrospective design inherently carries risks of selection bias and unmeasured confounding. As further demonstrated by the formal minimum sample size assessment according to the Riley framework, the relatively limited number of recurrence events may have affected model stability and contributed to variability in variable importance rankings; therefore, SHAP-derived feature contributions should be interpreted cautiously and validated in larger, more representative cohorts, and formal sensitivity analyses under alternative missingness assumptions were not performed (11). This limitation is particularly relevant to tumor necrosis and STAS, which were missing in 71.2% and 38.5% of patients with recurrence, respectively; consequently, their apparent predictor importance and fold-level stability should also be interpreted cautiously. Although the use of nested cross-validation was intended to mitigate the risk of overfitting, the use of random undersampling in the primary Random Forest model represents an additional methodological consideration, as it discards a proportion of majority-class observations during training and may influence model operating characteristics. The external validation cohort was intentionally balanced by outcome to provide an initial assessment of discrimination. Consequently, it was not suitable for assessment of calibration or absolute risk prediction and may be susceptible to spectrum bias. Validation in larger, consecutive external cohorts reflecting the natural recurrence prevalence is therefore warranted. While the primary analysis employed a binary endpoint, the observed recurrence proportion should not be interpreted as the definitive cumulative incidence of postoperative recurrence in this population. Because a proportion of patients had not yet completed the biological risk window during which recurrence may occur, the binary outcome should be interpreted as recurrence status at the last available follow-up rather than definitive lifetime recurrence-free status. We additionally performed complementary time-to-event analyses explicitly accounting for right censoring (see Time-to-Event Analyses Accounting for Censoring). Although these analyses are also influenced by the available duration of follow-up, they explicitly account for censoring and provide complementary evidence for interpreting the primary findings in the presence of heterogeneous follow-up and right censoring. A formal competing-risks framework accounting for non-recurrence death was not feasible because cause-of-death information was incompletely recorded in the study database. Smoking history, cumulative smoking exposure (pack-years), and extent of surgical resection were not included among the candidate predictors of the primary model. These are recognized clinical factors associated with recurrence risk, and their omission should be considered when interpreting the model’s predictors, particularly the apparent importance of the FEV1/FVC ratio, whose interpretation is further limited by substantial missingness within the recurrence subgroup (57.7% missing; **Table 1**); a complete-case sensitivity analysis was not performed given the already limited number of recurrence events. Similarly, baseline comparisons between the recurrence and non-recurrence groups could not include sex, histological cell type, perioperative complications, or length of hospital stay because these variables were not available in a format permitting reliable outcome-stratified reporting at the time of this revision, limiting the completeness of the reported baseline profile. Future studies incorporating reliable cause-specific mortality data and larger multicenter validation cohorts may further strengthen the clinical applicability of these findings.

## Conclusion

This study suggests that postoperative recurrence risk after curative surgery for early-stage (stage I–II) non–small cell lung cancer is primarily associated with tumor-pathological characteristics. In contrast, the present analyses did not establish an incremental prognostic contribution from host-related factors, and such signals should therefore be regarded as exploratory and hypothesis-generating. An explainable Random Forest model based on routinely available clinical and pathological variables demonstrated promising discrimination in an independent case-control external validation cohort, suggesting its potential role as a complementary decision-support tool for risk stratification. Future studies with a larger number of recurrence events, more mature follow-up, complete outcome ascertainment, and prospective external validation should evaluate whether survival-based modeling approaches provide incremental value over threshold-based classification frameworks for postoperative recurrence prediction before consideration for routine clinical application.

## Data Availability

The raw data supporting the conclusions of this article will be made available by the authors, without undue reservation.

## Supplementary material

Supplementary Figure 1 Overview of the machine learning development and validation workflow. The framework included model development in 723 patients with Stage I–II NSCLC, nested cross-validation with embedded preprocessing and hyperparameter optimization, internal out-of-fold validation, independent external validation in 50 patients, and evaluation of discrimination, calibration, clinical utility, and model interpretability.
