# Supplementary material for: Externally validated explainable machine learning for postoperative recurrence prediction in early-stage NSCLC
Source: Front Oncol. 2026 Sep 11;16:1913341. doi: 10.3389/fonc.2026.1913341 (PMC13612256; doi:10.3389/fonc.2026.1913341)
Supplement: Supplementary file 1 [file Table1.docx]

**Supplementary Material**

*Externally Validated Explainable Machine Learning for Postoperative Recurrence Prediction in Early-Stage NSCLC*

# Supplementary Table 1. TRIPOD Checklist

*Completed in accordance with the TRIPOD Statement for studies developing and validating a multivariable prediction model (Collins et al., 2015). “Yes” indicates the item is fully reported; “Partial” indicates the item is addressed but incompletely; “No” indicates the item is not reported; “N/A” indicates the item is not applicable to this study.*

| **Section/Item** | **Item** | **Checklist Item** | **Reported?** | **Location in Manuscript** |
| --- | --- | --- | --- | --- |
| Title and abstract | 1 | Identify the study as developing and/or validating a multivariable prediction model, the target population, and the outcome to be predicted. | **Yes** | Title ("...Explainable Machine Learning Model for Postoperative Recurrence in Early-Stage NSCLC") |
|  | 2 | Provide a summary of objectives, study design, setting, participants, sample size, predictors, outcome, statistical analysis, results, and conclusions. | **Yes** | Abstract |
| Introduction | 3a | Explain the medical context and rationale for developing/validating the model. | **Yes** | Introduction, paragraphs 1–2 |
|  | 3b | Specify the study objectives, including whether the study describes development, validation, or both. | **Yes** | Introduction, final paragraph; Methods (Study Design) |
| Methods: Source of data | 4a | Describe the study design, source of data, and key dates, separately for development and validation. | **Yes** | Methods (Study Design and Patient Population) |
|  | 4b | Specify key study dates, including start/end of accrual and follow-up. | **Partial** | Follow-up duration reported; exact accrual start/end dates not specified |
| Methods: Participants | 5a | Specify eligibility criteria for participants. | **Yes** | Methods (Study Design and Patient Population: Stage I–II NSCLC, curative resection, neoadjuvant therapy excluded) |
|  | 5b | Describe settings and locations of data collection. | **Yes** | Title Page (institutional affiliations); Methods |
|  | 5c | Give details of treatments received, if relevant. | **Yes** | Methods (curative resection); surveillance protocol specified |
| Methods: Outcome | 6a | Clearly define the outcome predicted, including how and when assessed. | **Yes** | Methods (postoperative recurrence, radiologically/pathologically confirmed); surveillance schedule specified |
|  | 6b | Report whether outcome assessment was blinded to predictor information. | **No** | Not explicitly reported — retrospective outcome ascertainment via chart review; blinding not applicable/stated |
| Methods: Predictors | 7a | Clearly define all predictors, including how and when measured. | **Yes** | Methods (Model Development); routinely available preoperative/pathological variables |
|  | 7b | Report whether predictor assessment was blinded to outcome data. | **No** | Not explicitly reported |
| Methods: Sample size | 8 | Explain how the study size was arrived at. | **Yes** | Methods (Validation Strategy — Minimum Sample Size Assessment); retrospective cohort of available eligible patients |
| Methods: Missing data | 9 | Describe how missing data were handled, with reasons. | **Yes** | Methods (Data Preprocessing and Missing Data Management) |
| Methods: Statistical analysis | 10a | Describe how predictors were handled in the analyses. | **Yes** | Methods (Model Development) |
|  | 10b | Specify type of model, all model-building procedures, and method for internal validation. | **Yes** | Methods (Model Development; Validation Strategy — Nested Cross-Validation) |
|  | 10c | For external validation, describe how the predictions were calculated. | **Yes** | Methods (Validation Strategy); Results (External Validation Performance) |
|  | 10d | Specify all measures used to assess model performance. | **Yes** | Methods (Model Performance, Calibration, and Clinical Utility) |
| Methods: Risk groups | 11 | Provide details on how risk groups were created, if done. | **Partial** | Youden-optimal threshold (0.41) reported for binary classification; formal risk-group stratification (e.g., tertiles) not performed |
| Methods: Development vs. validation | 12 | For validation, identify differences from development in setting, eligibility, outcome, predictors. | **Yes** | Discussion (Limitations); external cohort intentionally balanced by outcome |
| Results: Participants | 13a | Describe flow of participants, including numbers with/without outcome and, if applicable, a diagram. | **Partial** | Results (Patient Characteristics and Follow-up); no formal STROBE-style flow diagram included |
|  | 13b | Describe characteristics of participants, including predictor and outcome information. | **Yes** | Results (Patient Characteristics and Follow-up); Supplementary Table (baseline characteristics) |
|  | 13c | For validation, show comparison with development data. | **Partial** | External cohort composition described (n=50, balanced 25/25); direct side-by-side baseline comparison table not included |
| Results: Model development | 14a | Specify number of participants and outcome events in each analysis. | **Yes** | Results (n=723, 52 events for primary analysis; n=715, 44 events for time-to-event analyses) |
|  | 14b | Report unadjusted association between each candidate predictor and outcome, if done. | **No** | Not reported; variable importance reported via SHAP and permutation importance instead of univariable association statistics |
| Results: Model specification | 15a | Present the full prediction model to allow predictions in new individuals. | **No** | Not presented in a directly applicable equation/coefficient form, consistent with the non-parametric nature of the Random Forest model; SHAP summary (Figure 4) provided as an interpretability substitute |
|  | 15b | Explain how to use the model. | **Partial** | Youden-optimal threshold and predicted-probability interpretation described; a standalone user-facing tool/nomogram not provided |
| Results: Model performance | 16 | Report performance measures for prediction, with confidence intervals. | **Yes** | Results (Model Performance — Internal Validation; External Validation Performance; Calibration Analysis; Clinical Utility) |
| Results: Model updating | 17 | Report results from any model updating, if done. | **N/A** | No model updating performed in this study |
| Discussion | 18 | Discuss limitations, including sources of potential bias and generalizability. | **Yes** | Discussion (Limitations paragraph, substantially expanded during this revision) |
|  | 19a | For validation, discuss results in relation to performance in development data. | **Yes** | Discussion; Results (External Validation Performance) |
|  | 19b | Give an overall interpretation considering objectives, limitations, and results from similar studies. | **Yes** | Discussion |
| Discussion: Implications | 20 | Discuss potential clinical use and implications for future research. | **Yes** | Discussion, concluding paragraphs |
| Other: Supplementary information | 21 | Provide supplementary resources (study protocol, statistical code, datasets). | **Partial** | Title Page: data available from authors upon request for verification; statistical code not publicly deposited |
| Other: Funding | 22 | Provide funding source and role of funders. | **Yes** | Title Page ("This research received no specific grant from any funding agency...") |

# Supplementary Table 2. PROBAST Risk-of-Bias Assessment

*Completed in accordance with the PROBAST tool (Wolff et al., 2019) for studies developing and validating a multivariable prediction model. Domain-level judgements are reported transparently, including domains rated at high risk of bias, rather than citing PROBAST completion alone as a general marker of methodological rigor without accompanying documentation.*

| **Domain** | **Signaling Questions (summarized)** | **Risk of Bias Judgement** | **Rationale** |
| --- | --- | --- | --- |
| **1. Participants** | Was appropriate data source used (e.g., cohort/RCT)? Were inappropriate exclusions avoided? | **Low** | Single-country, two-institution retrospective cohort with clearly defined, clinically justified eligibility criteria (Stage I–II NSCLC, curative resection, neoadjuvant therapy excluded); no evidence of inappropriate exclusions. |
| **2. Predictors** | Were predictors defined and assessed in a similar way for all participants? Were predictors available at the time the model is intended to be used? Were predictors assessed without knowledge of outcome data? | **Low** | Predictors were routinely available preoperative and pathological variables, consistently defined and measured across the cohort; predictor assessment temporally precedes outcome ascertainment. |
| **3. Outcome** | Was the outcome determined appropriately? Was a prespecified/standard definition used? Was the outcome defined/determined without knowledge of predictors? Was the time interval between predictor assessment and outcome determination appropriate? | **High** | The primary analysis retained a binary outcome definition susceptible to bias from heterogeneous follow-up and informative censoring (recurrence rate 7.2% versus 20–35% expected from cited literature). Complementary time-to-event analyses were performed to explore the impact of censoring but did not replace the binary framework used for the primary model, calibration, decision-curve analysis, and SHAP interpretability. |
| **4. Analysis** | Were there a reasonable number of participants with the outcome (events per variable)? Were continuous/categorical predictors handled appropriately? Were all enrolled participants included in the analysis? Were participants with missing data handled appropriately? Was selection of predictors based on univariable analysis avoided? Were complexities in the data (e.g., censoring) accounted for appropriately? Were relevant model performance measures evaluated appropriately? Was overfitting/optimism accounted for? | **High** | A formal minimum sample size calculation (Riley et al., 2020) indicated the development cohort provided only approximately 13–29% of the recommended minimum number of events for the candidate predictor sets considered. Random undersampling was used to address class imbalance, altering natural outcome prevalence and further reducing the effective training sample. These factors, combined with the limited number of recurrence events (n=52), constitute a high risk of overfitting and unstable performance/variable-importance estimates, as reflected in the fold-level SHAP stability analysis. |

**Overall risk of bias judgement:** High. This overall judgement is driven by the Outcome and Analysis domains and is consistent with the study's framing throughout the manuscript as a hypothesis-generating, methodologically transparent analysis requiring larger, prospectively validated cohorts before clinical application.

**Supplementary Table 3. PROBAST Applicability Assessment**

| **Domain** | **Concern regarding applicability** | **Judgement** | **Rationale** |
| --- | --- | --- | --- |
| **1. Participants** | Do included participants match the review question / target population? | **Low** | Population (resected Stage I–II NSCLC) matches the intended target population for a postoperative recurrence-risk tool. |
| **2. Predictors** | Do predictor definitions/timing match the intended use setting? | **Low** | Predictors are routinely available at the time of postoperative risk assessment in comparable clinical settings. |
| **3. Outcome** | Does the outcome definition match the intended use? | **Low-to-moderate** | Postoperative recurrence is the clinically relevant outcome; residual concern relates to outcome ascertainment under heterogeneous follow-up (see Analysis/Outcome domains above). |

**Overall applicability judgement: Low-to-moderate concern. The study population and predictors align well with the intended clinical use case; residual concern relates to the outcome ascertainment issues detailed above and the artificially balanced external validation cohort, which limits generalizability of absolute risk estimates pending validation in a consecutive, naturally-prevalent cohort.**

# Supplementary Table 4. Time-to-Event Model Performance (Bootstrap 95% Confidence Intervals)

*Harrell’s concordance index (C-index) and 36-month time-dependent area under the receiver operating characteristic curve (AUC), with 95% bootstrap confidence intervals (1,000 resamples of pooled out-of-fold predictions), for six Cox proportional hazards and Random Survival Forest (RSF) model configurations evaluated on the time-to-event cohort (n = 715; 44 recurrence events).*

| **Model** | **Variable set** | **C-index (95% CI)** | **36-month time-dependent AUC (95% CI)** |
| --- | --- | --- | --- |
| Cox | TNM-only | 0.66 (0.57–0.73) | 0.63 (0.54–0.71) |
| RSF | TNM-only | 0.60 (0.51–0.68) | 0.57 (0.48–0.67) |
| Cox | Parsimonious | 0.66 (0.58–0.73) | 0.63 (0.54–0.72) |
| RSF | Parsimonious | 0.64 (0.55–0.72) | 0.61 (0.52–0.71) |
| Cox | Expanded | 0.58 (0.49–0.66) | 0.54 (0.44–0.64) |
| RSF | Expanded | 0.66 (0.57–0.73) | 0.63 (0.53–0.71) |

**References for this Supplementary Material**

Collins GS, Reitsma JB, Altman DG, Moons KG. Transparent reporting of a multivariable prediction model for individual prognosis or diagnosis (TRIPOD): the TRIPOD statement. Ann Intern Med. 2015;162(1):55–63.

Wolff RF, Moons KGM, Riley RD, et al. PROBAST: A Tool to Assess the Risk of Bias and Applicability of Prediction Model Studies. Ann Intern Med. 2019;170(1):51–58.

Riley RD, Ensor J, Snell KIE, et al. Calculating the sample size required for developing a clinical prediction model. BMJ. 2020;368:m441.
